# An economic analysis of interseeding of pasture and feeding legume forages to dairy cows in an organic system

**DOI:** 10.3168/jdsc.2025-0968

**Published:** 2026-05-20

**Authors:** Marielle E. Mumford, Joleen C. Hadrich, Andre F. Brito, Bradley J. Heins

**Affiliations:** 1 Department of Applied Economics, University of Minnesota, St. Paul, MN 55108; 2 Department of Agriculture, Nutrition, and Food Systems, University of New Hampshire, Durham, NH 03824; 3 Department of Animal Science, University of Minnesota, St. Paul, MN 55108; 4 West Central Research and Outreach Center, University of Minnesota, Morris, MN 56267

## Abstract

This study assessed the economic viability of interseeding a legume-grass mixture in an existing organic pasture system, focusing on the implications for milk yield, milk composition, and profitability. Using net present value analysis, financial and production data were analyzed from the University of Minnesota Farm Financial FINBIN database and university feeding trials (University of Minnesota and University of New Hampshire). The analysis modeled a baseline 88-cow organic dairy herd with an average milk yield of 7,185 kg. A 10-yr net present value analysis was used to account for the time value of money at a discount rate of 7% and fluctuating pasture productivity, with interseeding occurring in years 1, 5, and 10. The analysis found a positive net present value of $394,274, which indicated that the investment was financially feasible for organic dairy farms. Sensitivity testing found that although the modeled farm was resilient to increased seed costs, profitability was more dependent on milk price and production. Specifically, an 18.7% decrease in milk price or a reduction in annual milk yield to 5,844 kg/cow would be required to reduce the net present value to zero. In conclusion, integrating legume-grass mixtures into organic rotational grazing systems was economically feasible and could support long-term profitability.

## Highlights

• Interseeding legume-grass mixes in organic dairy systems was financially viable.
• A 10-year analysis showed a positive NPV exceeding $394,000.
• Farm profitability remained robust even if legume seed costs doubled or tripled.
• Success depended more on milk price and production than on pasture seed costs.
• Organic legume pastures support long-term economic and environmental health.

## Graphical Abstract

This study evaluated the financial impact of interseeding legume-grass mixtures into organic rotational grazing systems for dairy cows. Using a 10-year net present value (NPV) analysis, an 88-cow organic dairy herd with an average milk yield of 7,185 kg was modeled. The results indicated that this transition was highly profitable and found a positive NPV of $394,274. Sensitivity analysis indicated that the dairy farm's profitability was resilient to significant increases in seed costs; however, it remained sensitive to fluctuations in milk prices and production. These results provide organic dairy farmers with information that investing in diverse, legume-mixed pastures was a sound financial decision that supported long-term farm viability while utilizing the natural nitrogen fixing benefits of legumes to reduce reliance on external inputs.

**Figure:**
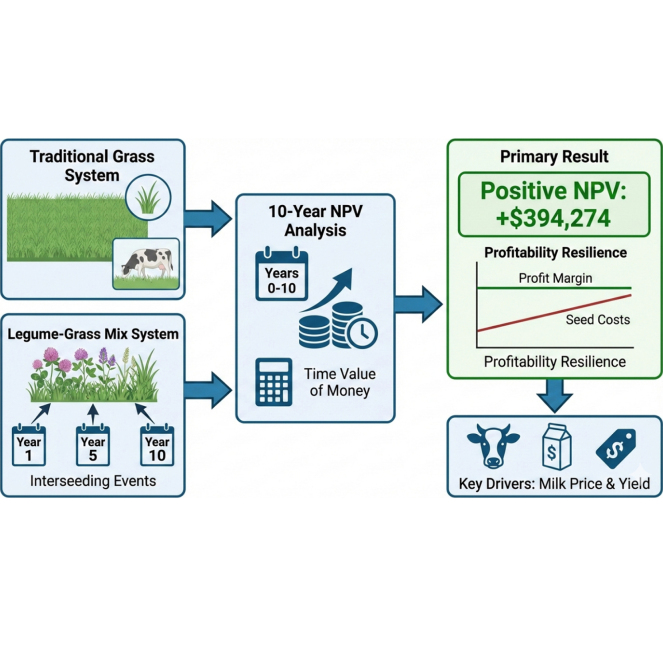

The distinction between organic and conventional dairy farming has been a topic of considerable interest within the dairy industry, particularly in relation to milk composition, milk yield, and environmental impact. Organic dairy production is characterized by the prohibition of synthetic growth hormones, antibiotics, and genetically modified organisms (**GMO**), as well as reliance on grazing of pasture and organic feed sources (USDA-NIFA, 2012). In contrast, conventional dairy farming uses synthetic fertilizers, pesticides, and high-energy feed inputs to enhance milk production efficiency (USDA, 2015). Whereas conventional systems typically have higher milk yields, organic systems that rely heavily on a grass-based diet are often associated with improved milk nutritional quality and a lower environmental impact (Gomiero et al., 2011; Benbrook et al., 2018).

One of the primary nutritional benefits attributed to organic milk is its increased concentration of n-3 fatty acids, which are essential for human health and have been linked to reduced risks of cardiovascular disease and improved cognitive function (Ruxton et al., 2004; Swanson et al., 2012). These elevated levels of n-3 fatty acids in organic milk are largely due to the higher proportion of pasture-based forage in organic dairy cow diets, particularly from legume-rich pastures (Benbrook et al., 2018). Furthermore, organic milk has been found to have a more favorable n-6 to n-3 ratio compared with conventional milk, a factor that has been suggested to enhance health benefits (Benbrook et al., 2018).

Beyond nutritional differences, milk yield is another key area of difference between organic and conventional dairy systems. Organic dairy cows typically have lower milk yields, and organic grazing systems demonstrated the greatest reduction in output compared with TMR-based feeding systems (Roesch et al., 2005; McBride and Greene, 2010; Schwendel et al., 2015). Compared with conventional confinement systems, organic grazing herds frequently have smaller body size and crossbred cows selected with more emphasis on improved fertility, productive life, and forage efficiency relative to milk production (Ahlman et al., 2011, 2014). For pasture-based dairy herds, Holstein cows generally have highest milk production but tend to have longer calving intervals, greater body condition loss, and lower fertility compared with other breeds. In contrast, Jersey, Montbéliarde, Normande, Norwegian Red, and various crossbreds typically have improved fertility, better body condition, and greater productive life with similar fat plus protein production (Washburn and Mullen, 2014). The lower milk production in organic systems is attributed to the reliance on high-forage diets with limited access to concentrated energy sources, in contrast to conventional systems where TMR feeding enables higher nutrient intake and increased milk yield.

Despite lower milk yields, organic dairy farmers benefit from price premiums that help offset production costs. Organic milk consistently commands higher retail prices compared with conventional milk, reflecting consumer demand for perceived health and environmental benefits (Smith et al., 2009). However, transitioning from conventional to organic dairy production presents economic and managerial challenges, including increased feed and labor costs, certification expenses, and production constraints (McBride and Greene, 2010; MacDonald, 2020). Furthermore, environmental sustainability is a significant factor influencing dairy production systems. Organic dairy farms generally have lower GHG emissions than conventional systems due to reduced reliance on synthetic nitrogen fertilizers and increased utilization of legume-based pastures for nitrogen fixation (Bos et al., 2014; Muller et al., 2017). This practice contributes to enhanced soil fertility while mitigating nitrogen loss to water and associated environmental concerns (Gomiero et al., 2011).

Assuming that profitability gains must come from increased milk production is inconsistent with established pasture-based dairy economics. Grazing systems frequently generate profit through reduced input costs and higher efficiency of pasture harvest rather than through maximized milk production per cow (Neal and Roche, 2019). Organic grazing systems often rely on cows selected for forage efficiency and moderate milk production rather than maximizing production, which may influence the economic response to pasture-based management strategies. Therefore, this study evaluated whether legume interseeding may improve long-term economic returns through more uniform pasture productivity across a grazing season and system efficiency, independent of changes in milk production.

The objective of this study was to evaluate the economic feasibility of interseeding legume-grass mixtures into existing organic rotational grazing systems using a 10-yr net present value framework. The hypothesis was that integrating legumes into organic pasture systems would generate positive long-term economic returns due to improved forage productivity, without negatively affecting milk production. Furthermore, it was expected that profitability would be more sensitive to milk price and production levels than to seed or interseeding costs. This study did not seek to compare grazing systems directly with TMR organic systems; rather, the focus was to determine whether pasture renovation within an organic grazing system represents a financially sound capital investment.

This study evaluated the economic return of incorporating a legume-grass mixture into an organic rotational grazing system for an 88-cow Holstein dairy herd producing an average of 7,185 kg of milk per cow. Assessing this multiyear investment required consideration of both pasture productivity changes and cost differences associated with establishing and maintaining legume-based pastures, as well as their effects on milk production. Because pasture establishment represents a long-term decision on grazing farms, a net present value (**NPV**) approach was used to account for the time value of money and to incorporate changes in pasture productivity and associated cash inflows and outflows over time (Kay et al., 2008; Barry and Ellinger, 2012).

An investment in a capital asset is considered financially viable when the expected return exceeds the cost of capital. Under NPV, an investment should be made if NPV is greater than zero, indicating that projected returns exceed costs on a discounted basis. If NPV equals zero, the decision-maker is indifferent, as the investment is expected to exactly cover its opportunity cost. Conversely, if NPV is less than zero, the investment should be rejected because anticipated returns do not fully compensate for the cost of capital. The NPV calculation is defined as

[1] $$\begin{array}{c} \mathrm{NPV}=\\ -Investment_{t=0}+\frac{\sum_{t=1}^{T=10}\left(Cash\;Inflows_{t}-Cash\;Outflows_{t}\right)}{{\left(1+\mathrm{WACC}\right)}^{t}}, \end{array}$$

where *Investment* is the interseeding investment of an existing pasture, *Cash Inflows_t_* are the revenues generated for the organic dairy farm including cull cow sales, calf sales, and milk revenue at time *t*, *Cash Outflows_t_* include cash operating expenses and fixed expenses from the dairy operation at time *t*, *T* is the net present value for each year, and the weighted average cost of capital (**WACC**) is the discount rate. The WACC considers the after-tax return to the capital asset, which is calculated from the farm's marginal tax rate, solvency measures from the balance sheet, as well as the cost of equity and cost of debt. This allows the decision-maker to observe the full impact of the after-tax implications of this investment decision.

The University of Minnesota West Central Research and Outreach Center (**WCROC**) organic dairy herd and the University of New Hampshire (**UNH**) organic dairy herd interseeded their pastures with a legume-grass mix as part of grazing research to evaluate if milk yield or quality changed with a legume-grass mix pasture. The legume-grass mix seed cost was recorded at each site with an average cost of $235.32/ha. The interseeding cost was valued at the inflation-adjusted price of $72.82/ha following Barnhart (1995). Total investment for interseeding was estimated at $308.14/ha. Economic assumptions were derived from University of Minnesota FINBIN farm financial database rotational grazing enterprise budgets, which likely reflect herds composed primarily of Holstein cows. Therefore, the modeled cost structure may be more representative of Holstein-based organic grazing herds than herds with smaller-framed breeds or crossbreds.

Legume pasture productivity was estimated at 1,367 kg/ha, which generated 53,404 kg of pasture forage on 35 ha in 120 d (Tozer et al., 2015; Ritz et al., 2020; Cartmill and Donaghy, 2024). Legume-grass mix pastures have been shown to have yield productivity decreases of 5% annually (Tozer et al., 2015; Ritz et al., 2020; Cartmill and Donaghy, 2024). The economic role of interseeding in the study was to preserve pasture forage availability across the grazing season and avoid greater reliance on purchased feed inputs. Lactating cows were assumed to consume 13.6 kg of DM per day of pasture with the remainder supplied through stored forages and supplemental feeds on annual herd level average. Therefore, 43,110 kg of legume pasture is needed to maintain the nutritional requirements of an 88-cow Holstein dairy herd with an average milk production of 7,185 kg of milk.

The primary data source for this study was the University of Minnesota FINBIN farm financial database, which aggregates financial information from thousands of farm operations in Midwestern states of the United States (FINBIN, 2025). The database provides enterprise budget reports that offer insight into average costs and revenues for various dairy production systems. Furthermore, additional data were acquired from the WCROC organic dairy herd (https://wcroc.cfans.umn.edu/research/dairy), the UNH organic dairy herd (https://colsa.unh.edu/facility/organic-dairy-research-farm), and the USDA Agricultural Marketing Service. These sources provided detailed insights into pasture renovation costs, seed pricing, and the economic factors influencing dairy production.

Economic data were from 2021 enterprise budget reports using FINBIN's sort feature for rotational grazing operations (FINBIN, 2025). The 2021 data year aligned with the initiation of grazing research trials at WCROC and UNH. Although both universities provided information on pasture seed costs, they were unable to supply comprehensive enterprise-level financial data necessary to conduct the NPV analysis. In addition, university-based expenses are not representative of typical organic dairy farm cost structures. Therefore, FINBIN data were used to generate more practical and broadly applicable financial estimates. The summary enterprise budget included 26 dairy farms and reflected the following average economic values.

Cash inflows included milk sold, dairy calves sold, cull sales, government payments, and insurance income associated with the dairy herd, which had 88 cows, on average. Those cows produced 7,185 kg of milk per year and received an average milk price of $0.618/kg. The total cash inflow per cow per year was $4,419.82 (FINBIN, 2025).

Cash outflows included operating and ownership costs. Operating expenses include feed, breeding fees, supplies, fuel and oil, repairs, hired labor, utilities, hauling and trucking, marketing, bedding, and miscellaneous expenses. Total feed costs were $2,093.74/cow, with $100.27 attributed specifically to pasture. The remaining feed expense reflected corn grain, corn silage, alfalfa, haylage, protein and minerals, and other feedstuffs. These figures represent annual expenses rather than costs limited to the grazing season. All other operating expenses, excluding feed, averaged $1,020.23/cow (FINBIN, 2025).

Ownership expenses included management labor, depreciation, building leases, farm insurance and interest payments for existing loans associated with the organic dairy farm, and income taxes. Of the total ownership expenses ($658.56/cow), depreciation is a non-cash expense. Although depreciation ($200.61/cow) was not included in the cash outflow calculation for NPV, it was needed to calculate the income tax expense for the year because it provides a deduction and will decrease tax liability. All assumptions used for the NPV are included in Table 1.

**Table 1:** Economic, farm, and pasture productivity assumptions

Item	Number	Source
Farm assumption		
Number of cows	88	FINBIN, 2025
Milk yield, kg/cow	7,185	FINBIN, 2025
Pasture assumption		
Hectares needed per cow	0.405	
Legume pasture yield, kg/ha	1,367	Tozer et al., 2015; Ritz et al., 2020; Cartmill and Donaghy, 2024
DMI, daily kg	13.608	
DMI in pasture, kg	4.082	
Economic assumption		
Milk price, $/kg	0.618	FINBIN, 2025
Legume seed mix, $/ha	235.32	WCROC, UNH
Legume interseeding, $/ha	72.82	Barnhart, 1995
Total cash inflows, $/cow	4,419.82	FINBIN, 2025
Pasture feed expense, $/cow	100.27	FINBIN, 2025
All other feed expense, $/cow	1,993.47	FINBIN, 2025
Operating expense, $/cow	1,020.23	FINBIN, 2025
Ownership expense, $/cow	658.56	FINBIN, 2025
WACC, %	7	
Tax rate, %	35	
Inflation rate, %	2	

Microsoft Excel (version 16.66.1, Microsoft Corp.) was used to evaluate a NPV to analyze interseeding investment in organic rotational grazing pasture systems. The NPV model included the initial investment cost, cash inflows, cash outflows, and the associated farm characteristics that affected these estimates.

The NPV results are in Table 2 and reflect the sum of discounted cash inflows and outflows over the 10-yr analysis period. After-tax net cash flows were discounted annually using the WACC. For example, the after-tax net cash flow in yr 7 was discounted to present value (yr 0) using the formula $\mathrm{present}\mathrm{value}=\frac{\mathrm{net}\;\mathrm{after}-\mathrm{tax}\;\mathrm{cash}\;\mathrm{flo}w_{t=7}}{{\left(1.07\right)}^{7}}.$ This procedure was applied to each year of the investment horizon. The NPV reported in Table 2 represents the sum of these discounted annual after-tax net cash flows.

**Table 2:** Net present value of interseeding legume pasture

Item	Year										
	0	1	2	3	4	5	6	7	8	9	10
Initial investment	−10,978					−12,121					−13,382
Cash inflows, $		388,944	396,723	404,658	412,751	421,006	429,426	438,014	446,775	455,710	464,824
Cash outflows											
Pasture expenses, $		8,824	9,000	9,180	9,364	9,551	9,742	9,937	10,136	10,338	10,545
All other feed expenses, $		175,425	178,934	182,513	186,163	189,886	193,684	197,557	201,509	205,539	209,650
All other operating expenses, $		89,780	91,576	93,407	95,276	97,181	99,125	101,107	103,129	105,192	107,296
Ownership expenses, $		40,300	41,106	41,928	42,766	43,622	44,494	45,384	46,292	47,217	48,162
Depreciation expenses (non-cash), $		17,654	18,007	18,367	18,734	19,109	19,491	19,881	20,279	20,684	21,098
Income taxes, $		19,937	20,335	20,742	21,157	17,338	22,012	22,452	22,901	23,359	19,142
Total cash outflows, $		334,265	340,951	347,770	354,725	369,698	369,056	376,437	383,966	391,645	408,177
After-tax cash flows, $		54,679	55,772	56,888	58,025	51,308	60,370	61,577	62,809	64,065	56,648
Discounted after-tax cash flow, $	−10,978	51,086	48,684	46,395	44,214	36,526	40,154	38,266	36,466	34,752	28,709
NPV, $	394,274										

The 10-yr NPV for interseeding legume-grass pasture on a 5-yr cycle was positive. The pasture was interseeded at a cost of $308.13/ha in yr 0 for a total investment of $10,978. Pasture productivity decreased by 5% each year, and by yr 5, there would not be enough pasture yield to meet the DMI of the herd, so interseeding is needed. The investment cost of interseeding pasture increased to $12,121, and in yr 10 the total investment was $13,382. These investment costs were adjusted to future values to account for inflation and the change in the value of the dollar in the future. Although the final interseeding investment occurs at the end of the 10-yr period and its benefits are not fully realized within the modeled horizon, it represents a necessary reinvestment to maintain pasture productivity beyond the study period. Therefore, the yr 10 investment should be interpreted as positioning the farm for continued pasture productivity rather than as an investment fully recouped within the 10-yr time period. Results from the WCROC and UNH feeding trials indicated no change in milk yield nor milk composition, and therefore, total cash inflows remained at $4,419.82/cow, which resulted in annual cash inflow of $388,944 for the herd. This was increased annually with a 2% inflation rate.

Cash outflows in yr 1 were determined based on the per cow rate reported in Table 1 and multiplied by the number of cows in the herd for the total annual cash outflow. Income taxes were calculated on an annual basis, considering revenue, operating expenses, and non-cash expenses, such as depreciation. A change in pasture expenses was not observed as pasture productivity decreased because the initial pasture productivity generated close to 9,979 kg of additional forage over what was required.

Discounted after-tax cash flows were expected to decline over time because future dollars are worth less than dollars received today. Noticeable reductions in after-tax cash flow occurred in yr 5 and 10, which corresponds to additional interseeding investments. In those years, increased cash outflows reduced net cash flow; however, income tax liabilities declined due to the higher deductible expenses associated with the investment.

The positive NPV of $394,274 indicated interseeding of pasture is a worthwhile investment for organic dairy farms. However, NPV are sensitive to the assumptions, which are the foundation of the model. To test the sensitivity of the model, the legume-grass seed cost was increased to determine the price that makes the farmer indifferent between investing in interseeding pasture with a legume-grass mix versus choosing not to interseed. The legume seed cost was increased by 50% to $352.99/ha, which resulted in an NPV of $386,257. Doubling the seed price resulted in an NPV greater than $300,000. This demonstrated that the price of seed was not the determining factor in profitability for the organic dairy farm. The sensitivity of NPV to seed cost may differ if legume interseeding influenced milk yield, milk composition, or required greater pasture maintenance compared with traditional grass systems, as these factors would directly affect both revenues and operating costs. Knowing that the interseeding investment was small compared with the full costs of an organic dairy farm, milk production and milk price were evaluated to determine the impact of this decision. If the milk price decreased to $0.502 kg, the NPV would be zero. This would be an 18.7% decrease in the milk price received by organic dairy farmers. Although this price may be common for conventional dairy producers, it would be too low for an organic dairy farm to operate profitably (O'Hara and Parsons, 2013; Walsh et al., 2020). Similarly, milk production would need to decrease to 5,844 kg per cow per year to make the NPV equal to zero. The positive NPV observed in this study was consistent with previous whole-farm research that demonstrated that pasture-based dairy systems may improve profitability when milk organic premiums are maintained at high levels (O'Hara and Parsons, 2013; Walsh et al., 2020).

The current study demonstrated that investing in interseeding of a legume-grass system was economically feasible for organic dairy operations with rotational pasture grazing systems. Although the financial analysis demonstrated positive value, there were components of this decision that are not captured in the NPV analysis. Previous stochastic modeling studies have demonstrated that milk price variability, feed cost fluctuations, and weather-related forage variability substantially influenced dairy farm returns (Shalloo et al., 2004; Liang et al., 2017). Changing weather patterns makes it challenging to have an optimal window to interseed a new pasture grass or legume and give it enough time to mature before putting cows out in the pasture rotation. Furthermore, legume seed costs were higher than a traditional grass mix, and with no difference with milk production or composition, it may be difficult for a dairy farmer to justify making this management change without further productivity benefits, either at the milk production or pasture productivity level.

Legume-grass mixtures may also improve pasture resilience under changing climatic conditions. Mixed swards have been shown to enhance drought tolerance, forage stability, and seasonal yield distribution compared with monoculture grass systems (Tozer et al., 2015; Cartmill and Donaghy, 2024). Beyond direct financial returns, legume integration may provide ecosystem services including improved soil carbon sequestration, enhanced biodiversity, and reduced nutrient loss (Gomiero et al., 2011; Bos et al., 2014). Although these benefits were not monetized in the current analysis, emerging carbon markets and ecosystem service payment programs may increase the financial returns to legume-based pasture systems.

Herd genetics and cow body size may also influence the economic outcomes of grazing-based systems. Cows selected for grazing environments, including smaller-framed breeds (i.e., Jersey, Normande, and New Zealand Holstein Friesian) and crossbreds, typically have lower maintenance energy requirements and improved fertility, which can enhance the efficiency of converting pasture into milk. In contrast, larger-framed, high-producing Holstein cows may require greater supplemental feed to maintain production under grazing conditions. Therefore, the economic benefits of pasture improvements may be more fully realized in herds genetically adapted to grazing-based systems (Heins et al., 2008, 2011; Washburn and Mullen, 2014).

Previous forage research has shown that legume-grass mixtures can improve forage nutritive value and pasture persistence relative to grass monocultures (Hopkins and Del Prado, 2007; Muir et al., 2011). Although these factors may contribute to improved grazing performance, the current study did not include a direct comparison with traditional grass pastures. Therefore, the positive economic returns identified in this study are based on modeled financial outcomes and should not be attributed solely to changes in forage quality or persistence. Furthermore, whole-farm comparisons of pasture-based and confinement systems suggest differential cost structures reinforce the importance of sustainable forage management in organic dairying (Nehring et al., 2021). Although organic operations face higher feed and labor expenses, they often have greater profitability through premium milk prices and increased pasture reliance. Although external market premiums and ecosystem services were not included in the analysis, they represent areas for future research and could further enhance the economic viability of legume-based grazing systems.

In summary, the positive NPV over the 10-yr horizon suggested that pasture renovation with legumes may support long-term profitability without negatively affecting milk production. Although returns were sensitive to milk price and production levels, the investment in interseeding remained financially resilient under changes in seed cost. This study demonstrated that integrating legume forages into organic dairy systems can be profitable beyond the initial investment period. These findings highlighted the potential for long-term profitability in legume-based grazing systems, although farmers must carefully weigh the trade-off between an initial reduction in profit and the prospect of increased profitability in future years.
